# The use of telephone in genetic counseling versus in-person counseling: a randomized study on counselees’ outcome

**DOI:** 10.1007/s10689-012-9522-x

**Published:** 2012-03-08

**Authors:** Ulla Platten, Johanna Rantala, Annika Lindblom, Yvonne Brandberg, Gunilla Lindgren, Brita Arver

**Affiliations:** 1 Department of Oncology and Pathology, Karolinska University Hospital, 17176 Stockholm, Sweden; 2 Department of Clinical Genetics, Karolinska Institutet, 17176 Stockholm, Sweden; 3 Department of Oncology and Pathology, Karolinska Institutet, 17176 Stockholm, Sweden

**Keywords:** Hereditary cancer, Oncogenetic in-person counseling, Oncogenetic telephone counseling, Cancer worry, Oncogenetic nurse

## Abstract

Increased demand for genetic counseling services necessitates exploring alternatives to in-person counseling. Telephone counseling is a less time-consuming and more cost-effective alternative. So far there is insufficient evidence to support a pre-counseling telephone model. This randomized questionnaire study aims to evaluate the oncogenetic counseling process and to compare the impact of the initial part of the oncogenetic counseling, when conducted via telephone versus in-person. The aspects of evaluations were: patients’ expectations, satisfaction and experiences of genetic counseling, worry for developing hereditary cancer and health related quality of life. A total of 215 participants representing several cancer syndromes were randomized to counseling via telephone or in-person. The questionnaires were completed before and after oncogenetic nurse counseling, and 1 year after the entire counseling process. Overall, a high satisfaction rate with the oncogenetic counseling process was found among the participants regardless of whether the oncogenetic nurse counseling was conducted by telephone or in-person. The results show that a considerable number of participants experienced difficulties with the process of creating a pedigree and dissatisfaction with information on surveillance and prevention. Affected participants reported lower levels in most SF-36 domains compared to non-affected and both groups reported lower levels as compared to a Swedish reference group. The results indicate that telephone pre-counseling works as well as in-person counseling. Emotional support during genetic counseling and information on recommended cancer prevention and surveillance should be improved.

## Introduction

Studies on patients receiving genetic counseling at hereditary cancer clinics have to a large extent indicated high levels of satisfaction [1–5]. There are, however, areas of lesser satisfaction with oncogenetic counseling such as emotional support and understanding one’s own cancer risk [1, 3, 5, 6].

A number of publications discuss genetic counseling via telephone versus traditional in-person counseling. There is an agreement that in order to meet the increased demand for genetic services in general, and to gain better access in rural areas in particular, alternatives to in-person genetic counseling must to be explored [7–11]. Patient advantages with telephone versus in-person counseling could be immediate access to services, increased control over the interaction, privacy and anonymity [7] and avoidance of stress and expense [9, 12]. One disadvantage is lack of non-verbal cues [7]. From both the patient and the health-care point of view, costs can be reduced with telephone counseling [9, 13].

While post-result counseling has been tested and found to convey a high satisfaction level regardless whether it was conducted by telephone or in-person [13, 14], there is little evidence to support a pre-counseling telephone model [10, 11, 15].

This randomized questionnaire study aims at evaluating the counseling process in general at the department of Clinical Genetics at Karolinska University Hospital and to analyze the impact of the initial part of the counseling, the oncogenetic nurse counseling (OGNC), conducted via telephone versus in-person. The aspects of the evaluations are: patients’ expectations of genetic counseling, satisfaction and experiences with the different parts of the counseling process, worry for developing hereditary cancer and health related quality of life. The current study is part of a more comprehensive exploration and results from risk perception in the same cohort of participants have previously been published [16]. The overall intention of this study is to improve and facilitate the counseling process in order to meet patients’ needs.

## Materials and methods

### The standardized oncogenetic counseling procedure at the Karolinska University Hospital

Patients in the Stockholm County are referred to oncogenetic counseling by their general practitioners. The counseling process is divided into two parts. During the first part, the OGNC, patients are informed about the counseling procedure and familial cancer in general. A family pedigree is established in collaboration with the patient and cancer diagnoses in the family are confirmed through medical records and/or death certificates. OGNC is given either via telephone or in-person. Both OGNC groups are pre-informed of the content of the discourse and of the scheduled time and all OGNC is performed without disturbances and with ample time. During the second part of the counseling process the patients have a personal meeting with a physician including a review of: the family history of cancer, risk estimation, possibility of genetic testing, surveillance and prevention. All patients receive a letter summarizing the outcome after the completed counseling process.

### Study procedure

All patients with no previous genetic counseling in the family referred to the Department of Clinical Genetics at the Karolinska University Hospital during the course of 1 year were eligible to participate in a questionnaire study. A total of 309 patients fulfilled the criteria and were consecutively randomized to OGNC via telephone or in-person 1:1. They received a letter with study information and an invitation to participate. The letter also included the first of three questionnaires and a time for OGNC either via telephone or in-person. Patients willing to participate and who responded to at least one questionnaire were included in the study (participants). The second questionnaire was sent to the participants after completion of OGNC and physician counseling, and the third questionnaire was distributed 1 year after completing the entire counseling process. The questionnaires were sent together with a prepaid return envelope. The questionnaire data was collected during a study period of 3 years. One and the same nurse (UP) conducted all OGNC whereas seven physicians were involved in the second part of the counseling process during the study period.

### Study and questionnaire design

The questionnaires were designed jointly by a geneticist (AL), a psychologist (YB), an oncogenetic nurse (UP) and a graduate student of public health (GL). The questionnaires were tested on four patients and found feasible for the study. No formal reliability testing or validation was performed. The participants were given dissimilar questions regarding worry for cancer depending on whether or not they were previously diagnosed with the type of cancer they had a family history of i.e., “affected” or “non-affected”.

#### The first questionnaire (before OGNC)

The participants were asked to indicate:

1. On whose initiative they were referred to genetic counseling: their own, a physician’s, a relative’s or somebody else’s initiative. More than one response alternative to this item could be chosen.
2. The reason why they attended genetic counseling. The question was addressed as an open-ended answer query.
3. Their expectations of genetic counseling. The question was addressed as an open-ended answer query.

#### The second questionnaire (after OGNC)

The participants were asked to evaluate:

1. Their satisfaction with the counseling provided by the oncogenetic nurse on a four- level scale, from satisfied to a “very high extent”, “high extent”, “low extent” to “not at all satisfied”.
2. Their experience with the process of creating a pedigree. Five items were stated: interested in gaining knowledge of their family, positive to make contact with relatives, difficult to get information about relatives, the process took too long and the emotional difficulty in exposing themselves and their family. The participants were to use the four-level scale described above.
3. Their satisfaction regarding contact with the staff at the oncogenetic clinic. Seven items were to be evaluated on the four-level scale described above. The items were: being listened to, being understood, receiving answers to questions, taking part in decision making, emotional support, care provided and trust for the staff.

#### The third questionnaire (1 year after OGNC)

The participants were requested to evaluate:

1. Their satisfaction regarding contact with the staff at the oncogenetic clinic. See question nr 3 above in questionnaire nr 2.
2. Their experience of information and recommendations received. Four items were to be evaluated on the four-level scale described above. The items were: understanding of information, having received information on recommended surveillance and prevention, satisfaction with genetic counseling, and if genetic counseling could be recommended to relatives.

In all three questionnaires participants were to indicate their level of worry for developing cancer (non-affected) or developing cancer again (affected). The participants responded by using a check-box scale ranging from 1 to 7, from “not at all worried” to “extremely worried”.

The Short Form-36 (SF-36) Health Survey was used to measure the quality of life in participants in all three questionnaires [17]. SF-36 is composed of 36 items constituting eight health domains related to quality of life (HRQoL). The three dimensions measuring physical health are: physical functioning (PF, 10 items), role limitations due to physical problems (RP, 4 items) and bodily pain (BP, 2 items). The three mental dimensions are: mental health (MH, 5 items), role limitations due to emotional problems (RE, 3 items) and social functioning (SF, 2 items). The two well-being dimensions are: vitality (VT, 4 items) and general health (GH, 5 items). The SF-36 participants’ scores were compared to a gender and age-matched normative population sample (n = 215) formed on the basis of data from a Swedish population sample (n = 8,930). Application of this procedure resulted in the reference values for the population sample presented as mean value and standard deviation for each of the dimensions. The purpose of the SF-36 in the present study is threefold: to compare the quality of life between non-affected and affected participants; to compare the quality of life between the two OGNC groups and to determine the impact of time after genetic counseling on the participants’ quality of life.

### Ethical aspects

The study was performed in accordance with Swedish law (2003:460) and approved by the local Ethics Committee, D:nr 2005/566-31/1.

### Statistical analysis

Data analyses were performed with the Statistica 9 and SPSS program. Descriptive statistics were generated to display the participants by socio-demographic (age, gender) and medical variables (affected or non-affected) and for reporting the number of individuals with mean value, standard deviation (SD) and range. Differences between non-participants and participants were evaluated using, Mann–Whitney test for continuous variables (age) and Pearson’s exact χ^2^ test for categorical variables (gender and cancer status). Pearson’s exact χ^2^ test was also used to test difference in satisfaction and experiences of the pedigree process and in experience of information and recommendations. Binary logistic regression analysis was used to predict effect of cofounders (age, gender and cancer status, type of counseling i.e. in-person or telephone and on whose initiative patients were referred to genetic counseling) in items regarding satisfaction and experience. Wilcoxon matched pair test was used to analyze differences in worry for cancer over time in related samples.

## Results

### Characteristics of participants and non-participants

**Fig. 1:**
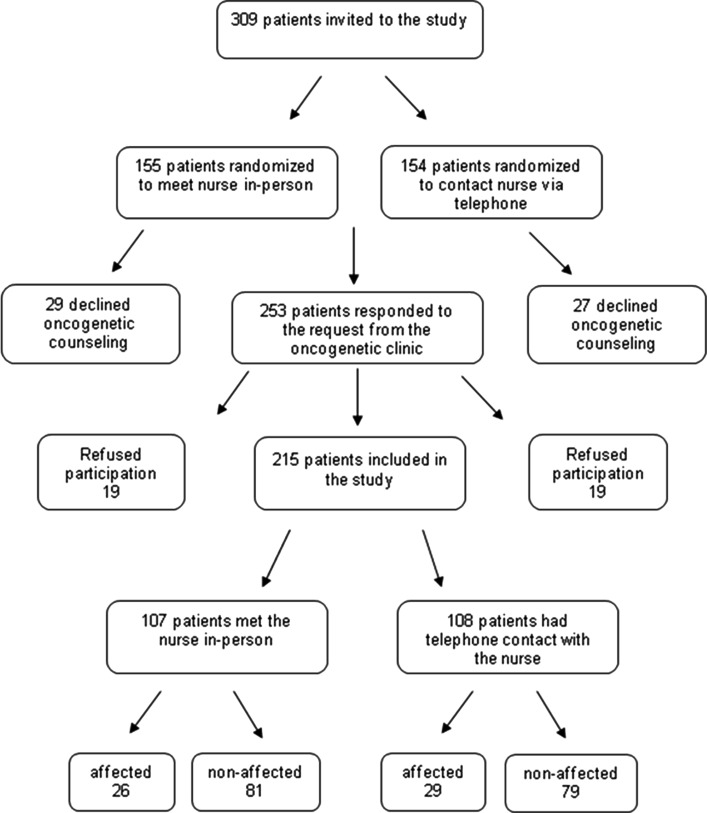
Flow chart of participant recruitment and retention

Among the referred patients 154 were randomized to OGNC via telephone and 155 to OGNC in-person (Fig. 1). A total of 28 patients from the first group and 29 from the second group declined genetic counseling. Of the remaining 253 patients, 19 in each group declined participation in the study (non-participants) and continued a standard genetic counseling procedure. A total of 215 patients (85% of eligible patients, n = 253), 107 from the in-person group and 108 from the telephone group returned at least one of the three questionnaires and were included in the study (participants) (Fig. 1).

A total of 99% (n = 213), (106 participants randomized to in-person and 107 to telephone OGNC), of the participants returned the first questionnaire. Two participants did not return the first questionnaire but returned the second and/or the third. The second questionnaire generated 156 responses (69 from the in-person OGNC group and 87 from the telephone OGNC group). The third questionnaire generated 141 responses (65 from the in-person OGNC group and 76 from the telephone OGNC group).

No significant differences were shown between participants (n = 215) and non-participants (n = 38) with respect to affected status, gender or age (Table 1).

**Table 1:** Cancer status, gender and age for participants and non-participants by group

	Affected	Non-affected	Female	Male	Age (SD) (Range)
Non-participants					
In-person (n = 19)	16% (n = 3)	84% (n = 16)	79% (n = 15)	21% (n = 4)	48 (9.9) (31-65) (n = 19)
Telephone (n = 19)	11% (n = 4)	79% (n = 15)	84% (n = 16)	16% (n = 3)	43 (14.4) (23-76) (n = 19)
All (n = 38)	18% (n = 7)	82% (n = 31)	82% (n = 31)	18% (n = 7)	46 (10.2) (23-76) (n = 38)
Participants					
In-person (n = 107)	24% (n = 26)	76% (n = 81)	91% (n = 97)	9% (n = 10)	45 (12.0) (16-71) (n = 107)
Telephone (n = 108)	27% (n = 28)	73% (n = 79)	88% (n = 95)	12% (n = 13)	45 (13.5) (20-87) (n = 108)
All (n = 215)	26% (n = 55)	74% (n = 160)	89% (n = 192)	11% (n = 23)	45 (12.8) (16-87) (n = 215)
	χ^2^ = 0.895, *p* = 0.344^a^		χ^2^ = 1.843, *p* = 0.175^b^		Z = 0.290, *p* = 0.772^c^

^a^Pearson’s Chi-square (*df* = 1) between all affected and non-affected, ^b^Pearson’s Chi-square (*df* = 1) between all females and males, ^c^Mann–Whitney to test age

The non-affected participants, 74% (n = 160), had a family history of: breast- (n = 104), ovarian- (n = 5), colorectal- (n = 43) endometrial- (n = 1) or gastric- (n = 3) cancer. The corresponding types of cancer among the affected participants, 26% (n = 55), were: breast- (n = 22), ovarian- (n = 10), colon- (n = 16), endometrial- (n = 1), gastric- (n = 1) and other- (n = 4) cancer. The familial cancer history of four non-affected and one affected participant was unknown, as they never attended genetic counseling after having answered the first questionnaire.

No significant difference was found between the two OGNC groups regarding affected status, types of cancer, age or gender. In both counseling groups the affected participants were significantly older than non-affected (*p* < 0.000). In the in-person counseling group the mean age of non-affected participants was 42.6 years (SD = 11.8, n = 81) and of the affected participants 52.7 years (SD = 9.5, n = 26). Corresponding figures for the telephone counseling group were 41.1 (SD = 12.0, n = 79) and 54.4 years (SD = 12.8, n = 29) respectively.

### Questionnaire one

#### Initiative to referral

A total of 51% (n = 110) of the participants were referred to oncogenetic counseling on physician’s initiative and 44% (n = 94) on their own, 6% (n = 13) a relative’s and 4% (n = 8) on “someone else’s” initiative. No statistical significant difference in initiatives of referrals was found between the two OGNC groups.

#### Reasons for and expectations of oncogenetic counseling

“A high frequency of cancer in the family” was the most common reason for attending genetic counseling (74%, n = 158). Other reasons were: “own cancer” (13%, n = 29) “anxiety” (11%, n = 24), “health problems” (10%, n = 21) and “for the sake of the family” (7%, n = 15).

The three most cited expectations of genetic counseling were “to obtain information about risk of developing cancer” (56%, n = 119), “to receive recommendations for preventive actions” (40%, n = 85) and “to estimate the risk for offspring” (15%, n = 33). Expectations were very similar independent of whose initiative patients were referred, but 11% (n = 12) of the patients who were “referred by a physician”, expressed no expectations regarding genetic counseling.

### Questionnaire two

#### Satisfaction regarding contact with the oncogenetic nurse

The participants, responding to this item, expressed overall high satisfaction regarding contact with the oncogenetic nurse: 94% (n = 144) were completely, 5% (n = 8) partially, 1% (n = 1) not very and 1% (n = 1) not at all satisfied. No statistical significant difference in satisfaction was shown regarding initiative (χ^2^ = 0.02, *p* = 0.877), gender (χ^2^ = 0.29, *p* = 592), affected status (χ^2^ = 0.75, *p* = 0.387), or between the two OGNC groups (χ^2^ = 0.02, *p* = 0.882).

#### Experience of the process of creating a pedigree

**Fig. 2:**
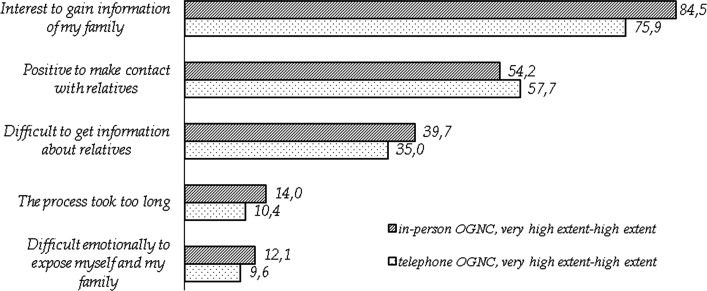
Experience with the process of creating a pedigree by group

Among participants, responding to this item, problems related to creating a pedigree were found. A total of 20% (n = 28) of all participants were not interested in gaining information about their family and 44% (n = 52) were not comfortable with contacting relatives (Fig. 2). Thirty-seven percent (n = 52) of the participants had difficulties in gaining information about relatives and 12% (n = 16) thought that the process of creating a pedigree took too long. Of the participants 11% (n = 15) expressed emotional difficulties in exposing themselves and their family.

No statistical significant differences in the experience of the process of creating a pedigree were found between the two OGNC groups. Age, gender, affected status or on whose initiative patients were referred to genetic counseling did not predict the outcomes.

### Questionnaire two and three

#### Satisfaction regarding contact with the oncogenetic clinic

**Fig. 3:**
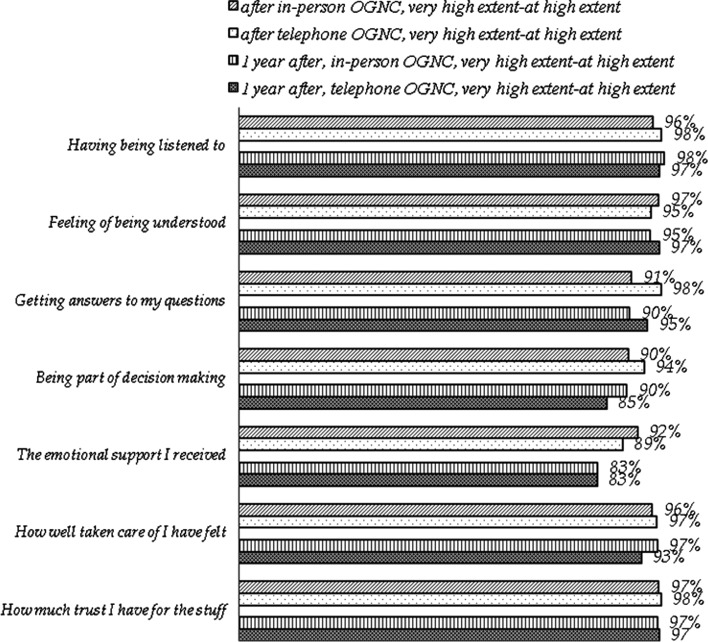
Satisfaction by group after counseling and at 1 year follow-up

At both assessment points, participants in both OGNC groups rated oncogenetic contacts as satisfying to a high extent (Fig. 3). No significant differences in satisfaction were found regarding OGNC group in the second or third questionnaire. Age, gender, affected status or on whose initiative patients were referred to genetic counseling did not predict the outcomes.

### Questionnaire three

#### Experience of information and recommendations

**Fig. 4:**
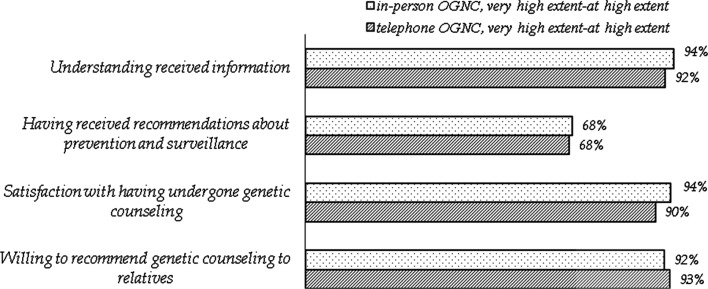
Experience related to information and recommendations 1 year after genetic investigations by group

In general, the participants responding to this item were satisfied with the information and with having undergone genetic counseling and they were willing to recommend genetic counseling to relatives. They were least satisfied with “having received recommendations on cancer prevention and surveillance” (Fig. 4.) A total of 32% (n = 43) were not satisfied. The type of OGNC, affected status, age, gender or on whose initiative the participants were referred did not predict the outcomes on any of the items.

### Worry of developing cancer

No significant difference in cancer worry between participants in the two OGNC groups was shown (Table 2). In both OGNC groups, non-affected participants’ cancer worry decreased during the study period. The reduction in cancer worry was significant between the first and the second assessment point (*p* < 0.005, *t* = 3.13) No difference was found between the second and the third point of assessment. In both OGNC groups, affected participants showed no significant difference in cancer worry between the first and second, or between the second and the third assessment point.

**Table 2:** Cancer worry before and after counseling and 1 year after genetic investigations by cancer status by group

	Before OGNC	After OGNC	p^a^, z^c^	1 year after	p^b^, z^c^
In-person counseling					
Non-affected participants	4.8 (n = 74)	4.0 (n = 50)	*p* < 0.005 z = -2.98	3.9 (n = 50)	*p* = 0.576 z = -0.58
Affected participants	5.2 (n = 26)	5.0 (n = 16)	*p* = 0.786 z = -0.43	5.4 (n = 15)	*p* = 0.500 z = -0.82
Telephone counseling					
Non-affected participants	4.8 (n = 73)	4.1 (n = 63)	*p* < 0.005 z = -4.2	3.8 (n = 59)	*p* = 0.547 z = -0.61
Affected participants	4.9 (n = 23)	5.1 (n = 14)	*p* = 0.217 z = -1.38	4.7 (n = 15)	*p* = 0.845 z = -0.23

p^a ^Between before genetic counseling and immediately after OGNC

p^b^ Between immediately after OGNC and 1 year after genetic counseling process

z^c ^Wilcoxon matched pairs

### SF-36

**Fig. 5:**
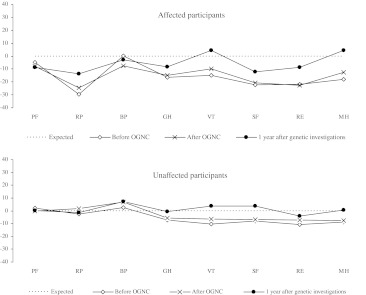
SF-36 scores by cancer status before and after genetic counselling and 1 year after genetic investigations

The affected participants reported lower levels in most of the SF-36 domains compared to non-affected at all three points of assessment (Fig. 5). No statistical significant differences in HRQoL were found between the two OGNC groups.

Both affected and non-affected participants reported lower levels in most of the domains as compared to a Swedish reference group. The reported scores in all domains improved over time for all participants and almost reached the level of the reference group.

## Discussion

When designing this study there was a preconception that patients might prefer and benefit from traditional in-person OGNC versus telephone OGNC. The results, however, provide no support for this presumption as no differences between the two methods of counseling were found. In both post-counseling questionnaires all investigated areas: participants’ expectations, satisfaction and experiences of genetic counseling, worry for developing hereditary cancer and quality of life according to SF-36 showed equal outcomes in both groups. Over time a high satisfaction rate was demonstrated in the two groups.

Interestingly 1 of 10 participants who were referred on the initiative of their physician did not have any expectations regarding genetic counseling, suggesting that more pre-counseling education may benefit these patients.

Creating a pedigree is the initial part of the oncogenetic counseling process where the participants have an active role in collecting information from relatives. Our study indicates that almost half of the participants found it difficult to contact relatives and a fifth did not appreciate learning about their families. One in ten of the participants found it emotionally difficult to expose themselves and their family and may have benefited from increased emotional support from the caregivers. This is in accordance with other studies stating that 20-40% of the patient desire more emotional support during the counseling process [1, 5]. Further, our study indicates that the dissatisfaction with the lack of emotional support was most pronounced among the young non-affected participants and with those diagnosed with low or moderate risk of developing hereditary cancer.

Information on recommended preventive actions seems to be an area for improvement in oncogenetic counseling, since as many as 33% of non-affected and 26% of affected participants were dissatisfied with information on advice and recommendations. Again, our results indicate that the dissatisfaction was most pronounced among non-affected with low or moderate risk of developing cancer. In addition to cancer specific surveillance, patients often ask for advice concerning lifestyle factors affecting cancer risk. However, lack of scientific evidence on risk reducing lifestyle factors makes it difficult to meet this expectation.

In congruence with a study from Pieterse et al. [5], the levels of cancer worry in affected participants remained constant during the study period, while in non-affected the levels declined. Cancer worry was not dependent on the mode of counseling i.e. telephone or in-person counseling. Risk perception over time did not differ between the OGNC groups, supporting equal comprehensive outcomes between groups (data not shown).

A weakness in our study is that genetic testing was not a variable. Genetic testing was performed in a minority of the participants and might have influenced the outcomes. However, in a previous study by Arver et al. [18] psychosocial effects of pre-symptomatic testing for breast/ovarian and colon cancer predisposing genes were evaluated. With the exception of the vitality domain of SF-36, no statistical differences between carriers and non-carriers were found in that study. Therefore, outcome of genetic testing was not a variable in the current study.

When comparing participants’ data with reference data, we found lower scores among participants in most of the domains in the SF-36 questionnaire before OGNC. This probably indicates that the family situation with cancer disease and worry of increased risk for cancer influence participants’ HRQoL. The participants may also be anxious about the outcome of the investigations. The scores improved over time and possible explanations are that participants processed and defused their anxiety through tools gained during the counseling process. Due to administrative failure, the last page of SF-36 Health Survey was missing in two-thirds of the third questionnaire, making the results for domains GH, VT, SF and MH difficult to interpret, since only 28 responses were obtained from the total of 141 participants responding to the last questionnaire.

In total only 15% of the referred participants declined participation in the questionnaire study, indicating a highly motivated population. Non-participants were equally distributed between telephone and in-person OGNC, speaking in favor of an unbiased cohort.

One of the strengths of this study is that one and the same oncogenetic nurse conducted both telephone and in-person OGNC and that this part of the counseling process was well defined. Another important strength of the study is that a validated health survey scale SF-36 was used. A third important aspect is that several cancer syndromes are represented in participants which increases applicability to clinical patient groups. One limitation however is that it is difficult to fully separate participants satisfaction with the OGNC from satisfaction with the entire genetic counseling process as questionnaire number two and three were distributed after OGNC *and* physician counseling. The use of questionnaires, developed to be study specific, but not tested for reliability or validity is another limitation. However, the questionnaires were tested for feasibility in a small sample of patients.

## Conclusions

Overall, a high satisfaction rate with the oncogenetic counseling process was found among the participants regardless of if the oncogenetic nurse counseling was conducted by telephone or in-person.

Given the results of our study, the option of a pre-counseling telephone model could be an equal or even better alternative to in-person counseling and could very well be a standard mode in the future. The results show that a considerable number of participants experienced difficulties with the process of creating a pedigree and dissatisfaction with information on recommended surveillance and prevention. These items should be areas of improvement.

## References

[1] Bleiker EMA, Aaronson NK, Menko FH (1997). Genetic counseling for hereditary cancer: a pilot study on experiences of patients and family members. Patient Educ Couns.

[2] Nordin K, Liden A, Hansson M (2002). Coping style, psychological distress, risk perception, and satisfaction in subjects attending genetic counselling for hereditary cancer. J Med Genet.

[3] Bjorvatn C, Eide GE, Hanestad BR (2007). Risk perception, worry and satisfaction related to genetic counseling for hereditary cancer. J Genet Counsel.

[4] Davey A, Rostant K, Harrop K (2005). Evaluating genetic counseling: client expectations, psychological adjustment and satisfaction with service. J Genet Counsel.

[5] Pieterse AH, Ausems M, Van Dulmen AM (2005). Initial cancer genetic counseling consultation: change in counselees’ cognitions and anxiety, and association with addressing their needs and preferences. Am J Med Genet Part A.

[6] Poeterse A, Van Dulmen S, Ausems M (2005). QUOTE-gene(ca): development of a counselee-centered instrument to measure needs and preferences in genetic counseling for hereditary cancer. Psycho-Oncology.

[7] Wang VO (2000). What is and is not telephone counseling?. J Genet Counsel.

[8] Ormond KE, Haun J, Cook L, Duquette D, Ludowese C, Matthews AL (2000). Recommendations for telephone counseling. J Genet Counsel.

[9] Sangha KK, Dircks A, Langlois S (2001). Assessment of the effectiveness of genetic counselling by telephone compared to a clinic visit. Am J Hum Genet.

[10] DeMarco TA, Smith KL, Nusbaum RH (2007). Practical aspects of delivering hereditary cancer risk counseling. Semin Oncol.

[11] Peshkin BN, Demarco TA, Graves KD (2008). Telephone genetic counseling for high-risk women undergoing BRCA1 and BRCA2 testing: rationale and development of a randomized controlled trial. Genet Test.

[12] Zilliacus EM, Meiser B, Lobb EA (2010). Women’s experience of telehealth cancer genetic counseling. J Genet Counsel.

[13] Jenkins J, Calzone KA, Dimond E (2007). Randomized comparison of phone versus in-person BRCA1/2 predisposition genetic test result disclosure counseling. Genet Med.

[14] Baumanis L, Evans JP, Callanan N (2009). Telephoned BRCA1/2 Genetic Test Results: Prevalence, Practice, and Patient Satisfaction. J Genet Counsel.

[15] Helmes AW, Culver JO, Bowen DJ (2006). Results of a randomized study of telephone versus in-person breast cancer risk counseling. Patient Educ Couns.

[16] Rantala J, Platten U, Lindgren G et al (2009) Risk perception after genetic counseling in patients with increased risk of cancer. Hereditary Cancer in Clinical Practice 7 10.1186/1897-4287-7-15 PMC2744911 19698175

[17] Sullivan M, Karlsson J (1994) Svensk manual och tolkningsguide (Swedish manual and interpretation guide). International Quality of Life Assessment (IQOLA) Project 2000

[18] Arver BHA, Platten U, Lindblom A, Brandberg Y (2004). Evaluation of psychosocial effects of pre-symptomatic testing for breast/ovarian and colon cancer predisposing genes: a 12-month follow-up. Fam Cancer.

